# Pathogenic roles of microRNAs and competing endogenous RNAs in Hirschsprung disease (HSCR): Potential diagnostic markers for HSCR

**DOI:** 10.1002/pdi3.21

**Published:** 2023-07-31

**Authors:** Lian Hou, Quan Kang

**Affiliations:** ^1^ Department of General and Trauma Surgery Children's Hospital of Chongqing Medical University National Clinical Research Center for Child Health and Disorders Ministry of Education Key Laboratory of Child Development and Disorders Chongqing China

**Keywords:** ceRNAs, enteric nervous system, enteric neural crest cells, Hirschsprung disease, microRNAs

## Abstract

MicroRNAs (miRNAs) are endogenous small non‐coding single‐stranded RNAs. They can bind to the 3′‐untranslated region (3′UTR) of mRNAs and regulate the expression of their target genes by inducing degradation or translation inhibition of the corresponding mRNAs. Competing endogenous RNAs (ceRNAs) can disable miRNAs by combining miRNAs response elements (MREs) with miRNAs. Hirschsprung disease (HSCR) is a common pediatric surgical disease in which cells derived from the enteric neural crest fail to colonize the distal colon, but its pathogenesis is not very clear. In recent years, with more and more studies on miRNAs in HSCR, miRNAs seem to be involved in the pathogenesis of HSCR. miRNAs in HSCR affect the proliferation and migration of enteric neural crest cells mainly through target genes, and ceRNAs inhibit miRNAs, thus participating in the pathogenesis of HSCR. It was reported that some miRNAs in the serum of children with HSCR were significantly higher than those in the control group. Therefore, miRNAs are expected to be a new noninvasive early screening biomarker and targeted therapy point for HSCR. Here, we provide a summary of the understanding of miRNAs and ceRNAs in regulating enteric nervous system proliferation and migration and their roles in the pathogenesis of HSCR.

## 1 INTRODUCTION

Hirschsprung disease (HSCR) is a common congenital pediatric surgical disease with genetic predisposition. The cumulative incidence of HSCR is approximately 1/5000,^1^ and the disease is 4 times more common in males than females.^2^ HSCR is characterized by the absence of intestinal ganglion, which always involves the internal anal sphincter and extends proximally, and the loss of intestinal neurons leads to tetanic contraction of the affected segments, resulting in a constricted and narrow intestine. Based on the length of the intestine lacking nerve cells, the illness is divided into three types: short segment HSCR (S‐HSCR), long segment HSCR (L‐HSCR), and total colonic ganglion necrosis.^3^ Symptoms of HSCR include constipation, bloating, and weight loss. The treatment of HSCR is surgical resection of ganglio‐enterostomy and colostomy. The pathogenesis of HSCR is extremely complicated and is currently poorly known. Genetic studies have demonstrated that a variety of gene mutations are associated with the pathogenesis of HSCR, such as tyrosine kinase receptor ret proto‐oncogene (RET), endothelial endothelin 3 (EDN3), transcription regulatory genes SRY‐box transcription factor 10 (Sox10), paired‐like homeobox 2B (PHOX2B) and zinc finger E‐box binding homeobox 2 (ZEB2), etc. However, only about 50% of pedigreed cases and 20% of random cases can be attributed to mutations of these well‐known genes, such as RET.^4^, ^5^, ^6^, ^7^, ^8^, ^9^

**FIGURE 1:**
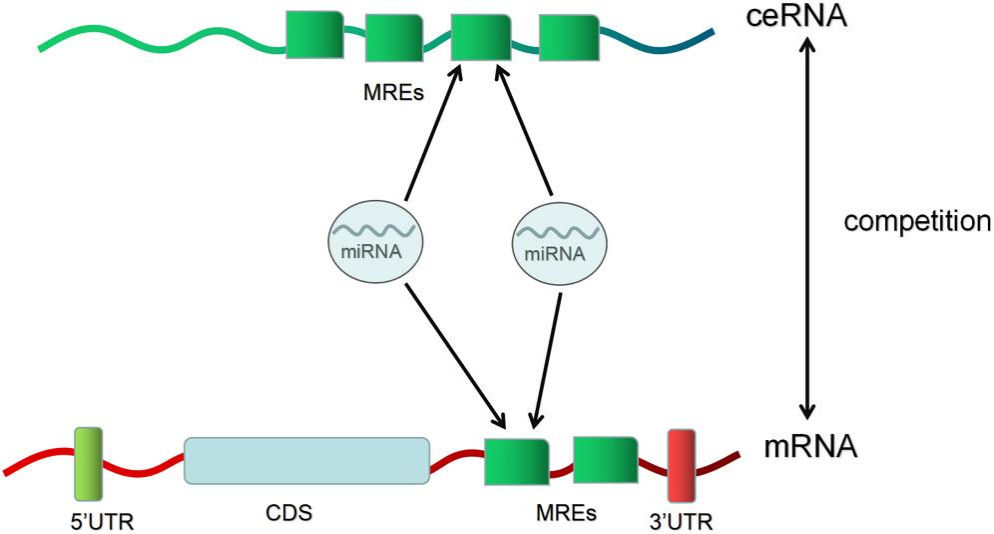
Mechanism of miRNAs and ceRNAs.

MicroRNAs (miRNAs) are endogenous small non‐coding single‐stranded RNAs. By causing the corresponding mRNAs to degrade or have its translation slowed down, they can attach to the 3′‐untranslated region (3′UTR) of mRNAs and control the expression of their target genes.^10^, ^11^ miRNAs play a role in many cellular functions, such as cell migration, proliferation, metabolism, and apoptosis.^12^, ^13^, ^14^, ^15^ miRNAs have potential application prospects in diagnosing, screening diseases, and exploring the pathogenesis of diseases, and have become a research hotspot in recent years. miRNAs can bind mRNAs to lead to gene silencing, while competing endogenous RNAs (CeRNAs) can bind miRNAs competitively to regulate gene expression, also known as miRNAs sponge. ceRNAs can disable miRNAs by combining miRNAs response elements (MREs) with miRNAs.^16^ However, there are not enough studies on ceRNAs in HSCR. This study will summarize the role of miRNAs and ceRNAs in the pathogenesis of HSCR (Figure 1).

## 2 MicroRNA GENE POLYMORPHISM CONTRIBUTE TO HSCR SUSCEPTIBILITY

Numerous research had examined the role of miRNAs polymorphisms in illness susceptibilities, including HSCR. Zhu et al.^17^ determined that the target gene Robo1, which is crucial in the pathophysiology of HSCR, may be downregulated due to rs2910164 polymorphisms in the pre‐Mir‐146a gene. In contrast to the CC genotype, Zheng et al.^18^ demonstrated that the CA/AA genotype of miR‐618 rs2682818 was associated with a lower incidence of HSCR. Based on the stratified analysis of HSCR subtypes, the rs2682818 CA/AA genotype significantly decreased the probability of HSCR in patients with long segments of HSCR compared to the CC genotype. The results showed that miR‐618 rs2682818 C>A polymorphism is associated with a decreased risk of HSCR in Chinese children, especially in patients with the long‐segment HSCR (L‐HSCR) subtype. Zheng et al.^19^ collected samples from 1473 control patients and 1470 HSCR patients. Real‐time fluorescence quantitative polymerase chain reaction was used to detect miR‐492 rs2289030 G>C TaqMan genotyping. The findings demonstrated that miR‐492 rs2289030 G>C polymorphism was not related to HSCR susceptibility in southern Chinese children. Zhong et al.^20^ discussed the link between miR‐938rs2505901 T>C polymorphism and the risk of HSCR in Chinese children. In comparison to the rs2505901 TT genotype, the rs2505901 TC and rs2505901 TC/CC genotypes were linked to a higher incidence of HSCR. In patients with the short‐segment HSCR subtype, the rs2505901 TC/CC genotype more strongly increased the advancement of HSCR. The findings demonstrated that the genetic variation of miR‐938rs2505901 influenced HSCR susceptibility and was connected to miR‐938 expression. Wu et al.^21^ conducted a stratified analysis of 1470 cases and 1473 controls and found that miR‐4318 rs8096901 polymorphism was associated with HSCR susceptibility in children in southern China, particularly in patients with short‐segment HSCR (S‐HSCR). Changes in HSCR susceptibility were linked to three positive 3′UTR variations in mitogen‐activated protein kinase10 (MAPK10). The 3′UTR cis‐acting regions of MAPK10 have a major impact on miR‐4516's ability to directly regulate the expression of MAPK10. In addition, the knockout of MAPK10 saved the effect of miR‐4516 on human neural cells migration. These findings suggested that HSCR risk was significantly influenced by miR‐4516 and its direct target MAPK10.^22^

## 3 THE PATHOGENESIS OF miRNA IN HSCR

### 3.1 miRNA downregulated target genes and inhibited the migration and proliferation of enteric neural crest cells

Many miRNAs affect the proliferation, differentiation, and migration of intestinal ganglion cells through corresponding target genes. Tang et al.^23^ detected the downregulation of miR‐141 expression in colon tissues of HSCR patients accompanied by increased expressions of CD47 and cullin 3 (CUL3) through real‐time quantitative polymerase chain reaction (PCR) and western blotting and confirmed the hypermethylation of CpG island in the promoter region of the miR‐141 gene. Using bisulfite sequencing polymerase chain reaction (BSP) analysis and verification by dual‐luciferase reporter assay, it was concluded that the downregulation of miR‐141 expression in colon tissues of HSCR patients induced an increase in the expression of its target genes, CD47 and CUL3, thus inhibiting the migration and proliferation of intestinal neural crest cells. Lei et al.^24^ detected that the expression level of miR‐195 in HSCR patients was significantly higher than that in the control group, and the expression of digestive organ dilatation factor (DIEXF) was decreased. A series of experiments confirmed that the expression of intracellular DIEXF was inhibited by increasing miR‐195, thus leading to the migration and proliferation of intestinal neural ridge cells to participate in HSCR. Li et al.^25^ suggested that the expression levels of miR‐200A and miR‐141 in HSCR patients were reduced by real‐time quantitative PCR and western blot, and dual‐luciferase reporter analysis showed that miR‐200A and miR‐141 could directly bind to the three ′non‐coding region of phosphatase and tensin homolog (PTEN), thereby inhibiting the expression of PTEN. The downregulation of MiR‐200A and miR‐141 inhibited the migration and proliferation of 293T and SH‐SY5Y cells by up‐regulating the expression of PTEN. Moreover, the inhibition of miR‐200A and miR‐141 on cell migration and proliferation was saved by knockout of the PTEN gene.

miR‐218‐1, miR‐206, miR‐192/215, miR‐24‐1*, miR‐369‐3p, miR‐483‐5p, miR‐142, miR‐483, miR‐214, miR‐431‐5p,miR‐140‐5p, miR‐195‐5p, miR‐92a, miR‐31/31*, miR‐770‐5p, and miR‐1251 were also involved in the pathogenesis of HSCR mediated by the regulation of target genes by miRNAs to inhibit the proliferation and migration of enteric neural crest cells (ENCCs) (Table 1).

**TABLE 1:** microRNAs in the pathogenesis of HSCR.

Author	Cohorts	Results	Conclusions
Tang et al.^26^	Colon tissues from 69 HSCR patients and 49 controls	Increased levels of MiR‐218‐1 and Slit2 and decreased levels of RET and PLAG1 mRNA and protein in colon tissues of HSCR patients	The regulation of Slit2, RET, and PLAG1 by MiR‐218‐1 may be involved in the pathogenesis of HSCR
Sharan et al.^27^	Tissues from 80 patients with HSCR and 80 controls without HSCR	The expression of miR‐206 was down‐regulated in tissues of HSCR cases	miR‐206 may play an important role in the pathogenesis of HSCR and inhibit cell migration and proliferation in disease models by targeting SDPR
Zhu et al.^28^	80 HSCR patients and 77 normal colon tissues	MIR‐192/215 was significantly down‐regulated in HSCR tissue samples	Down‐regulation of MiR‐192/215 mediates HSCR by inhibiting cell migration and proliferation through NID1
Tang et al.^29^	70 HSCR samples as compared with 74 normal controls	ARP2 and ARP3 were decreased in HSCR samples due to the increase of miR‐24‐1* and let‐7a*, respectively	The miR‐24‐1*/let‐7a*‐ARP2/3 complex‐Rac subtype pathway may represent a novel pathogenic mechanism of HSCR
Lei et al.^30^	70 HSCR patients and 62 normal colon tissues	The expression level of MiR‐215 in colon tissues of HSCR patients was significantly decreased, which was positively correlated with IARS2 expression and negatively correlated with SIGEC‐8 expression. miR‐215 inhibits SIGEC‐8 by directly binding to the 3 ′‐UTR of SIGEC‐8	miR‐215 acted synergistically with host gene IARS2 to affect the migration and proliferation of neurons through target gene SIGEC‐8
Pan et al.^31^	60 HSCR bowel tissue samples and 47 matched controls	The expression of miR‐369‐3p in HSCR tissues was significantly up‐regulated, which was negatively correlated with the decreased expression level of Sox4 gene. Dual‐luciferase reporter assay showed that the 3 ′‐UTR of Sox4 was a direct target of miR‐369‐3p	In SH‐SY5Y and 293T cells, HSCR was mediated through the abnormal expression of miR‐369‐3p and Sox4 genes, which significantly inhibited cell proliferation and migration
Wang et al.^32^	20 HSCR patients and 20 normal colon tissues	The expression of miR‐483‐5p in HSCR tissues was significantly increased. GFRA4 was the downstream target of miR‐483‐5p and was negatively correlated with the expression of miR‐483‐5p in cell lines	miR‐483‐5p may play a key role in the pathogenesis of HSCR by targeting GFRA4
Peng et al.^33^	48 HSCR aganglionic tissues and 48 normal bowel tissues	LPS is increased in HSCR tissues, and the increased LPS activates ADAR2, which subsequently regulates the editing of A‐to‐I RNA and inhibits the expression of miR‐142	LPS mediates HSCR through LPS‐ADAR2‐miR‐142‐STAU1 axis
Zhi et al.^34^	60 HSCR aganglionic colon tissues and 60 normal controls	miR‐483‐3p and its host gene IGF2 were found to be down‐regulated in HSCR ganglion‐free colon tissue. FHL1 was identified as the target gene of miR‐483‐3p by dual‐luciferase reporter assay	miR‐483‐3p derived from IGF2 is associated with Hirschsprung's disease by targeting FHL1 and may provide a new way to understand the etiology of HSCR
Wu et al.^35^	Colon tissues from 20 controls without HSCR and 24 patients with HSCR	The expression of miR‐214 was up‐regulated in HSCR tissue samples, and miR‐214 could directly down‐regulate PLAGL2	miR‐214 was involved in the pathophysiological processes of HSCR and inhibited cell proliferation and migration by directly down‐regulating PLAGL2 in cell models
Hu et al.^36^	Colon tissues of the HSCR group (*n* = 8) and the control group (*n* = 8)	The expression of miR‐431‐5p was up‐regulated and the expression of LRSAM1 was down‐regulated in ENCCs of HSCR. Luciferase assay confirmed that LRSAM1 was the target gene of miR‐431‐5p	Up‐regulation of miR‐431‐5p inhibits ENCCs proliferation by targeting LRSAM1
Du et al.^37^	Stenotic and dilated sections of the colon of 32 HSCR patients	miR‐140‐5p was significantly down‐regulated in HSCR tissue samples with increased EGR2 expression, and the expression of EGR2 in cell lines was negatively correlated with miR‐140‐5p	Down‐regulation of miR‐140‐5p may inhibit cell migration and proliferation by targeting EGR2 and promoting the pathogenesis of HSCR mediated by cell apoptosis
Wang et al.^38^	20 specimens from HSCR cases and 20 from matched control subjects	The expression of GFRA4 in HSCR colon tissue was significantly downregulated. GFRA4 is a direct target of miR‐195‐5p	MiR‐195‐5p plays an important role in the pathogenesis of HSCR by inhibiting nerve cell proliferation, invasion, and cell cycle arrest by targeting GFRA4 and accelerating nerve cell apoptosis
Ji et al.^39^	25 HSCR patients and 25 patients who were diagnosed without HSCR	In HSCR tissues, the expression of miR‐92a was increased, and the expression of KLF4 was decreased, and the expression was negatively correlated. KLF4 is the target of miR‐92a	miR‐92a inhibits proliferation and metastasis of nerve cells by regulating the KLF4/PI3K/AKT axis
Cai et al.^40^	Tissues samples from 90 HSCR patients and 90 matched controls	Downregulation of lncRNA MIR31HG in HSCR inhibited miR‐31 and miR‐31*. The downregulation of miR‐31 and miR‐31* enhanced inter‐α‐trypsin inhibitor heavy chain 5 (ITIH5) and the phosphatidylinositol‐4, 5‐bisphosphate 3‐kinase catalytic gamma subunit (PIK3CG)	MIR31HG‐miR‐31/31*‐ITIH5/PIK3CG pathway plays a role in the pathogenesis of HSCR
Li et al.^41^	96 HSCR patients and 96 controls	lncRNA MEG3 and intronic miR‐770‐5p decreased in HSCR patient samples. SRGAP1 was the target gene of miR‐770‐5p	The MEG3/miR‐770‐5p/SRGAP1 pathway plays a crucial role in the pathogenesis of HSCR
Zhou et al.^42^	32 HSCR patients and 32 controls	RMST and intronic MiR‐1251 were downregulated in the aganglionic colon of HSCR patients. Dual‐luciferase reporter assay was performed to confirm miR‐1251 targeted AHNAK	The novel RMST/SOX2/miR‐1251/AHNAK axis provided potential targets for the diagnosis and treatment of HSCR during the embryonic stage

Abbreviations: ADAR2, adenosine deaminase RNA specific; AKT, protein kinase B; ARP2/ARP3, actin related protein 2/actin related protein 3; EGR2, early growth response 2; ENCCs, enteric neural crest cells; FHL1, four and a half LIM domains 1; GFRA4, GNDF family receptor alpha 4; HSCR, Hirschsprung disease; IARS2, isoleucyl‐TRNA synthetase 2, mitochondrial; IGF2, insulin‐like growth factor 2; ITIH5, inter‐α‐trypsin inhibitor heavy chain 5; KLF4, Kruppel like factor 4; LPS, lipopolysaccharide; LRSAM1, leucine rich repeat and sterile alpha motif containing 1; MEG3, maternally expressed 3; MIR, microRNAs; NID1, nidogen 1; PIK3CG, phosphatidylinositol‐4, 5‐bisphosphate 3‐kinase catalytic gamma subunit; PLAG1, pleiomorphic adenoma gene 1; PLAGL2, PLAG1 like zinc finger 2; RMST, rhabdomyosarcoma 2 associated transcript; SIGEC‐8, sialic acid binding Ig‐like lectin 8; Slit2, slit guidance ligand 2; SDPR, serum deprivation‐response protein; Sox4, SRY‐Box transcription factor 4; SRGAP1, SLIT‐ROBO rho GTPase activating protein 1.

### 3.2 Role of ceRNA in HSCR

Some studies have explored the mechanism of ceRNA in HSCR. Chen et al.^43^ observed abnormal inhibition of lncRNA AFAP1 antisense RNA (AFAP1‐AS) in HSCR tissues. AFAP1‐AS knockout inhibited cell proliferation and migration and resulted in the loss of cell stress filament integrity, possibly due to AFAP1‐AS isolation of miR‐181a in HSCR cells. In addition, AFAP1‐AS down‐regulates RAS‐related protein 1b (RAP1B) via its ceRNA activity on miR‐181a. These results suggest that the abnormal expression of lncRNA AFAP1‐AS, a ceRNA of miR‐181a, may be involved in the occurrence and development of HSCR by enhancing RAP1B, the target gene of miR‐181a. Su et al.^44^ selected LOC100507600 from the gene expression chip data obtained from the intestinal tissues of HSCR patients and negative controls and verified the results in 64 pairs of HSCR‐induced intestinal stenosis tissues and negative controls by the qRT‐PCR method. It was found that LOC100507600 was significantly reduced in HSCR patient tissues and was significantly correlated with BMI1 proto‐oncogene, polycomb ring finger (BMI1). The direct effect of LOC100507600 on hsa‐miR128‐1‐3p and the competitive relationship between BMI1 and LOC100507600 were confirmed by dual‐luciferase reporter gene assay, protein extraction, and Western blotting. In addition, LOC100507600 down‐regulation inhibited cell migration and proliferation without affecting cell apoptosis and cycle.

Through the interaction between miRNA and ceRNA to regulate target genes to inhibit the proliferation and migration of intestinal nerve cells, the pathogenesis of HSCR was also mediated by miR‐1324, miR‐637, miR‐488‐3p, miR‐195, miR‐34a‐5p, miR‐148a‐3p,miR‐770‐5p, and the others (Table 2).

**TABLE 2:** ceRNAs in the pathogenesis of HSCR.

Author	Cohorts	Results	Conclusions
Zhou et al.^45^	48 HSCR aganglionic tissues and 48 normal bowel tissues	Circ‐PRKCI was significantly downregulated in HSCR ganglion tissue. Dual‐luciferase reporter assay and RNA immunoprecipitation assay confirmed the direct interaction between miR‐1324 and PLCB1 or circ‐PRKCI	As a molecular sponge of miR‐1324, circ‐PRKCI upregulated the expression of PLCB1 and inhibits cell proliferation and migration
Li et al.^46^	64 pairs of HSCR aganglionic tissues and matched normal tissues	FAL1 expression was downregulated in HSCR non‐ganglion tissue. FAL1 could positively regulate AKT1 expression by competitively binding to miR‐637	FAL1 may work as a ceRNA to modulate AKT1 expression via competitively binding to miR‐637 in HSCR
Wen et al.^47^	Control (*n* = 47); HSCR (*n* = 46)	Cirr‐CCDC66 expression in HSCR was downregulated. Cirr‐66 acts as a sponge for miR‐488‐3p to regulate DCX RNA expression	Cir‐CCDC66 modulates DCX expression through sponging miR‐488‐3p and thus participates in HSCR
Pan et al.^48^	42 pathological intestinal tissues from patients with HSCR and 42 normal intestinal tissues	lncRNA AFAP1‐AS1 levels were decreased in HSCR patients. When miR‐195 was upregulated, E2F3 was reduced, while the inhibition of AFAP1‐AS1 reduced its binding ability to miR‐195	AFAP1‐AS1 silencing acts as an endogenous RNA by interacting with miR‐195 to alter E2F3 expression, thus promoting HSCR progression
Sun et al.^49^	Tissues samples were obtained from 18 children with HSCR and 18 matched controls	lncRNA DRAIC in colon tissue of HSCR patients was significantly increased. A series of experiments demonstrated the competitive relationship between DRAIC and ITGA6	DRAIC regulated cell proliferation and migration by affecting the miR‐34a‐5p/ITGA6 signaling axis in HSCR
Li et al.^50^	30 aganglionic segment of the colon and 30 ganglionic segment of the colon	The expressions of LINC00346 and Dnmt1 in HSCR tissue were downregulated. miR‐148a‐3p inhibitors saved Dnmt1 downregulation in LINC00346 knockout cell lines	LINC00346 may be involved in the development of HSCR by regulating Dnmt1 expression as a ceRNA
Huang et al.^51^	Diseased and normal tissues from four patients with HSCR	Five circRNAs that were strongly up‐regulated in HSCR: hsa_circRNA_092493, hsa_circRNA_101965, hsa_circRNA_103118, hsa_circRNA_103279, and hsa_circRNA_104214	The network revealed a potential function of the circRNAs as molecular sponges targeting miRNAs and mRNAs in HSCR

Abbreviations: AFAP1‐AS1, AFAP1 antisense RNA 1; AKT, protein kinase B; DCX, doublecortin; Dnmt1, DNA Methyltransferase 1; DRAIC, downregulated RNA in cancer, inhibitor of cell invasion and migration; E2F3, E2F transcription factor 3; FAL1, focally amplified lncRNA on chromosome 1; HSCR, Hirschsprung diseaseITGA6, integrin subunit alpha 6.

### 3.3 Other roles of miRNA in HSCR

Some miRNA mediates HSCR through other ways rather than directly affecting the proliferation, migration, and differentiation of intestinal neural crest cells through their corresponding target genes or ceRNA participation. It has been shown that the SRY‐associated HMG‐box 9 (SOX9) is essential for the proper development of oligodendrocytes and astrocytes but not for the development of neurons. Quantitative polymerase chain reaction (QPCR), western blot analysis, and immunohistochemical detection showed that miR‐124 and its target gene, RY‐related HMG‐box 9 (SOX9) were overexpressed in the narrow colon segment of HSCR patients. These results suggest that HSCR may be related to the development of oligodendrocytes and astrocytes.^52^ Mir‐939 was significantly up‐regulated in HSCR tissues, and the expression of LRSAM1 was decreased. The direct association between miR‐939 and LRSAM1 was verified by the dual‐luciferase reporter assay. Overexpression of miR‐939 inhibited cell proliferation and did not affect apoptosis, cell cycle, or cell migration. LRSAM1 performs a similar function. Compared with the control group, the autophagy function of HSCR tissues was impaired. Although Mir‐939 reduced the expression of LRSAM1, it did not inhibit autophagy, and it is inferred that autophagy may also confer risk on HSCR.^53^

## 4 THE DIAGNOSTIC VALUE OF miRNAs IN HSCR

If without an early diagnosis, some patients with HSCR will develop serious complications such as toxic megacolon or HSCR‐associated enterocolitis. Therefore, it is particularly important to search for biomarkers of HSCR to assist in early diagnosis. Some studies have explored specific serum miRNAs or exosome miRNAs, which are expected to be the means of early, non‐invasive screening and diagnosis of HSCR.

Tang et al.^54^ collected serum samples from 95 HSCR cases and 104 matched controls. TaqMan low‐density array was used to initially screen for miRNAs expression. Candidate miRNAs were validated by individual reverse transcription real‐time quantitative PCR and two‐stage validation sets arranged in the training. In addition, to assess the diagnostic value and precision of serum miRNAs profiles in predicting HSCR, a double‐blind assay was also carried out in 23 patients with clinically suspected HSCR. Compared with the control group, five HSCR‐related miRNAs, including miR‐133a, miR‐218‐1, miR‐92a, miR‐25, and miR‐483‐5P, were identified using a multi‐stage assessment method. In the double‐blind test group, the 5‐miRNA profile's accuracy as a biomarker of HSCR was 82.6%, which was significantly better than the widely used contrast enema diagnostic method's accuracy of 70%. Exosomal miRNAs are more stable in blood and are more reliable biomarkers than miRNAs in plasma. Lv et al.^55^ collected plasma samples of HSCR patients and controls. Exosomal RNAs with aberrant regulation were obtained using high‐throughput Illumina sequencing after the exosomes had been extracted and further confirmed in two distinct cohorts. Bioinformatics analysis identified 31 abnormal miRNAs of which five were thought to be potential HSCR biomarkers. There were four significantly and steadily up‐regulated miRNAs (miR‐494‐3p, miR‐668‐3p, miR‐323a‐3p, and miR605‐3p) and one down‐regulated miRNA (miR‐5701), respectively. Hong et al.^56^ recently found that the levels of three plasma miRNAs (hsa‐miR‐192‐5p, hsa‐miR‐200a‐3p, and hsa‐miR‐200b‐3p) were higher than those of non‐HSCR patients through a series of experiments, which enriched the potential biomarkers of HSCR. By luciferase reporter and western blot analysis, it was established that these three miRNAs directly interact with the ZEB2 and fibronectin type III domain containing 3B (FNDC3B) target genes, decreasing cell viability and migration. miR‐199a‐3p suppresses cell growth and motility, partially by targeting mTOR. The expression of miR‐199a‐3p was upregulated in plasma exosomes of patients with HSCR. Plasma exosomal miR‐199a‐3p, a diagnostic marker, is crucial for the development of HSCR.^57^

## 5 DISCUSSION

miRNAs can not only play a regulatory function independently but also be linked to form an inter‐influencing ceRNA‐miRNA‐mRNA network. miRNAs bind to the MREs of mRNAs and lead to gene silencing. ceRNAs can disable miRNAs by combining MREs with miRNAs. miRNAs and ceRNAs are involved in the proliferation, migration, and differentiation of ENCCs and other cells in the gut, thereby mediating HSCR. These are exciting research topics for the future, as miRNAs and ceRNAs have potential application prospects in diagnosing, guiding disease staging, screening diseases, and exploring the pathogenesis of diseases. What is more, recent studies of HSCR in relation to miRNAs and ceRNAs offer promising avenues for the treatment of HSCR such as targeted therapy. However, the studies on the relationship between them are just the beginning, and more studies are needed to be explored. It is believed that with the further study, the mechanism of HSCR will be gradually clarified.

## AUTHOR CONTRIBUTIONS

Lian Hou collected materials and wrote the manuscript; Quan Kang contributed to the conception of the study.

## CONFLICT OF INTEREST STATEMENT

The author declares no conflict of interest.

## ETHICS STATEMENT

Not applicable.

## CONSENT FOR PUBLICATION

Not applicable.

## Data Availability

Data sharing is not applicable to this article as no datasets were generated or analyzed during the current study.
